# A novel replication-independent histone H2a gene in mouse

**DOI:** 10.1186/1471-2156-6-10

**Published:** 2005-02-19

**Authors:** Hiromi Nishida, Takahiro Suzuki, Yasuhiro Tomaru, Yoshihide Hayashizaki

**Affiliations:** 1 Laboratory for Genome Exploration Research Group, RIKEN Genomic Sciences Center (GSC), RIKEN Yokohama Institute, 1-7-22 Suehiro-cho, Tsurumi-ku, Yokohama, Kanagawa 230-0045, Japan

## Abstract

**Background:**

An uncharacterized histone H2a-coding transcript (E130307C13) has been cloned from a mouse full-length cDNA library. This transcript is encoded on chromosome 6, approximately 4 kb upstream of a histone H4 gene, *Hist4h4*. The proteins encoded by this transcript and the human *H2afj *mRNA isoform-2 have the highest amino acid similarity. In this paper, we characterize it from the expression pattern given by quantitative RT-PCR.

**Results:**

Quantitative RT-PCR indicated that the gene that encodes E130307C13 (*E130307C13*) is regulated in a replication-independent manner, and therefore it is *H2afj*. Certainly, *H2afj *transcript lacks a stem-loop structure at the 3'-UTR but contains a poly (A) signal. In addition, its promoter region has a different structure from those of the replication-dependent histone H2a genes.

**Conclusion:**

The bioinformatics imply that *E130307C13 *is a replication-independent H2a gene. In addition, quantitative RT-PCR analysis shows that it is replication-independent. Thus, it is *H2afj*, a novel replication-independent H2a gene in mouse.

## Background

Replication of the eukaryotic chromosomes requires the synthesis of histones to package the newly replicated DNA into chromatin. Control of the level of histone mRNA accounts for much of the control of histone protein synthesis [1]. Mouse has 18 replication-dependent histone H2a genes [2]. Among these 18 genes, 13 genes are located in the *Hist1 *cluster on chromosome 13, 4 in the *Hist2 *cluster on chromosome 3 and 1 in the *Hist3 *cluster on chromosome 11 [2]. The histone mRNAs that are cell-cycle-regulated increase 35-fold as cells progress from mitosis through G1-phase and into S-phase [3]. The promoters of histone genes contain CCAAT and TATA boxes [4]. The replication-dependent histone H2a genes lack introns and a poly (A) signal. They have a conserved stem-loop structure (5'-GGCTCTTTTCAGAGCC-3') at the 3'-UTR, which plays an important role in mRNA processing and stability [5-7].

Mouse also has two replication-independent histone H2a genes, *H2afx *on chromosome 9 and *H2afz *chromosome 3. These two genes encode polyadenylated mRNAs. *H2afx *mRNA has both a polyadenylated tail and a stem-loop structure [8]. Mouse replication-dependent histone H2a mRNAs and *H2afx *mRNA have a nuclear export element (5'-ACAACAAGAAGACGCGCATCAT-3') in the protein-coding region that functions to export the mRNA from the nucleus to the cytoplasm [9].

An uncharacterized histone H2a-coding transcript (E130307C13, FANTOM clone ID; NM_177688, Genebank accession number) has been cloned from a mouse full-length cDNA library. *E130307C13 *lies on chromosome 6, approximately 4 kb upstream of *Hist4h4*. In this paper, we characterize it from the expression pattern given by quantitative RT-PCR. In addition to this, we compared the structure with the sequences deposited in the international DNA/protein database.

## Results and discussion

**Figure 1:**
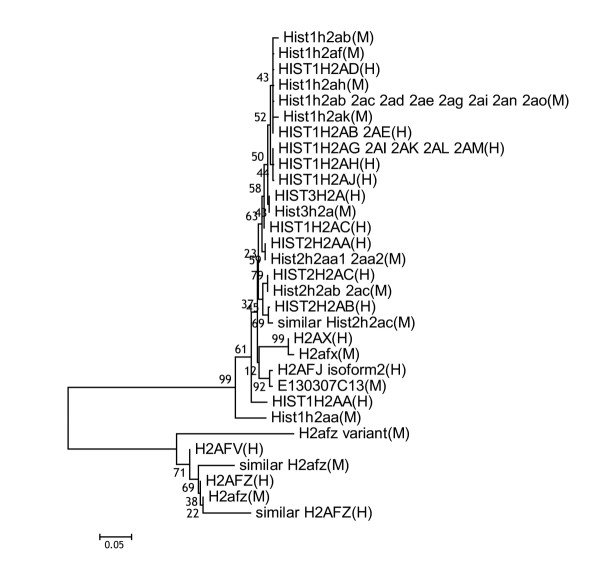
Phylogenetic relationships among 44 amino acids sequences from human and mouse histone H2a related proteins. The bar indicates 5% difference of sequence. The numbers at the branches indicate percentage of 1000 bootstrap analyses. (H) and (M) indicate the human sequence and mouse sequence, respectively.

**Figure 2:**
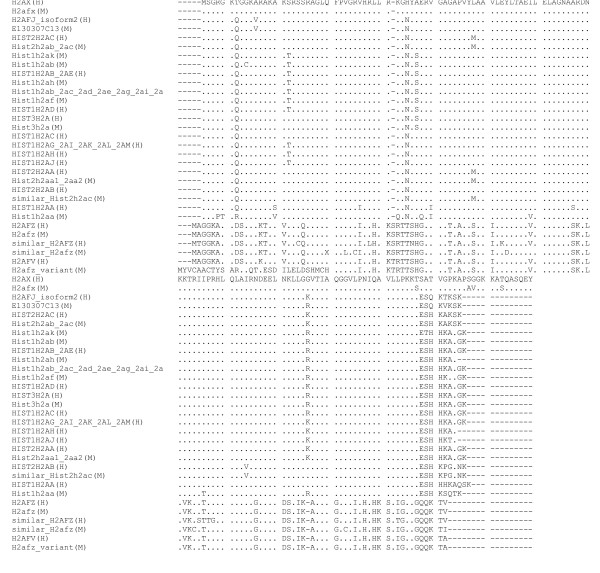
Alignment of the human and mouse histone H2a proteins in Fig. 1.

Comparison of the putative amino acid sequence encoded by *E130307C13 *with the amino acid sequences deposited in the international DNA/protein database showed that it has the highest similarity to that encoded by human *H2afj *mRNA isoform-2 (NM_177925, Genebank accession number; Figs. 1, 2). Human has two isoforms of *H2afj *[10,11]. Isoform-1 (NM_018267, Genebank accession number) is produced after splicing of two introns; isoform-2 does not need intron splicing for maturation. Interestingly, *H2afj *also lies near a histone H4 gene, on human chromosome 12. According to the nomenclature of histone genes [2], if *E130307C13 *is regulated in a replication-dependent manner, it is recognized as *Hist4h2a*. But if it is regulated in a replication-independent manner, it is recognized as *H2afj*.

**Figure 3:**
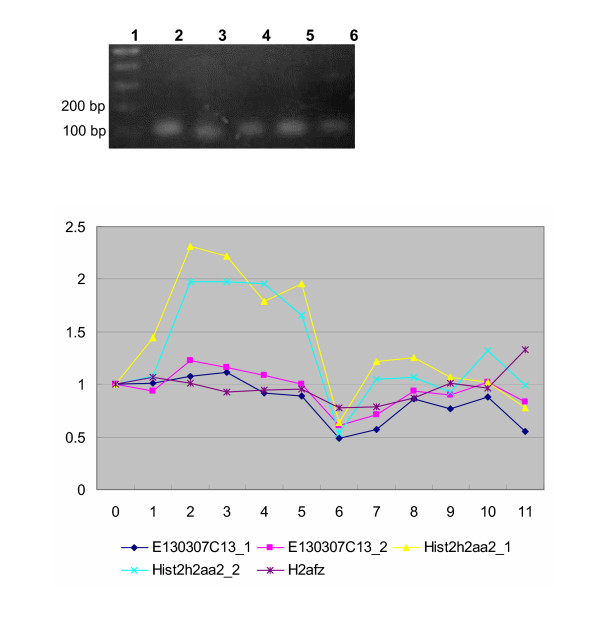
RT-PCR products on agarose gel and expression patterns. Lanes 1, 100 bp ladder; 2, RT-PCR product amplified with E130307C13 primer set 2; 3, that with E130307C13 primer set 1; 4, that with Hist2h2aa2 primer set 1; 5, that with Hist2h2aa2 primer set 2; 6, that with H2afz primer set.

Each product of the quantitative RT-PCR gave a single band on the agarose gel of the expected size (Fig. 3). Observation using the quantitative RT-PCR showed that the expression pattern of *E130307C13 *is typical of replication-independent histone gene (Table 1, Fig. 3). The expression pattern of *E130307C13 *is more similar to that of the replication-independent *H2afz *than to that of the replication-dependent *Hist2h2aa2*. The expression of *Hist2h2aa2 *increased along with cell cycle progression from the beginning of S-phase (0 h), peaked at 2 h, and then decreased (Fig. 3). On the other hand, *E130307C13 *and *H2afz *were expressed constantly (Fig. 3). These results suggest that *E130307C13 *is a replication-independent histone H2a gene in mouse. According to the nomenclature of histone genes [2], *E130307C13 *is recognized not as *Hist4h2a *but as *H2afj*.

**Table 1:** C_T _values, ΔC_T_, and ΔΔC_T_

Time (h)	GAPDH	E130307C13_1	ΔC_T_	ΔΔC_T_	Expression	E130307C13_2	ΔC_T_	ΔΔC_T_	Expression	Hist2h2aa_1	ΔCt	ΔΔC_T_	Expression	Hist2h2aa_2	ΔC_T_	ΔΔC_T_	Expression	H2afz	ΔC_T_	ΔΔC_T_	Expression
0	14.4	19.1	4.65	0	1	20	5.61	0	1	18.1	3.63	0	1	19.3	4.9	0	1	16.8	2.35	0	1
1	14.4	19	4.64	-0.01	1.01	20.1	5.71	0.1	0.93	17.5	3.1	-0.53	1.44	19.2	4.79	-0.11	1.08	16.6	2.26	-0.09	1.06
2	14.4	18.9	4.54	-0.11	1.08	19.7	5.32	-0.29	1.22	16.8	2.42	-1.21	2.31	18.3	3.92	-0.98	1.97	16.7	2.33	-0.02	1.01
3	14.3	18.8	4.5	-0.15	1.11	19.7	5.4	-0.21	1.16	16.8	2.48	-1.15	2.22	18.3	3.92	-0.98	1.97	16.8	2.46	0.11	0.93
4	14.4	19.2	4.78	0.13	0.91	19.9	5.49	-0.12	1.09	17.2	2.79	-0.84	1.79	18.3	3.93	-0.97	1.96	16.8	2.43	0.08	0.95
5	14.4	19.2	4.82	0.17	0.89	20	5.61	0	1	17.1	2.66	-0.97	1.96	18.6	4.17	-0.73	1.66	16.8	2.42	0.07	0.95
6	14.1	19.8	5.69	1.04	0.49	20.4	6.33	0.72	0.61	18.4	4.29	0.66	0.63	19.9	5.8	0.9	0.54	16.8	2.71	0.36	0.78
7	14.2	19.7	5.45	0.8	0.57	20.3	6.11	0.5	0.71	17.6	3.35	-0.28	1.21	19	4.83	-0.07	1.05	16.9	2.7	0.35	0.78
8	14.3	19.2	4.87	0.22	0.86	20.1	5.71	0.1	0.93	17.6	3.3	-0.33	1.26	19.2	4.81	-0.09	1.06	16.9	2.55	0.2	0.87
9	14.3	19.3	5.03	0.38	0.77	20	5.76	0.15	0.9	17.8	3.54	-0.09	1.06	19.3	5.01	0.11	0.93	16.6	2.34	-0.01	1.01
10	14.2	19.1	4.83	0.18	0.88	19.8	5.58	-0.03	1.02	17.8	3.6	-0.03	1.02	18.7	4.5	-0.4	1.32	16.6	2.4	0.05	0.97
11	14.3	19.8	5.5	0.85	0.55	20.2	5.87	0.26	0.84	18.3	4	0.37	0.77	19.2	4.91	0.01	0.99	16.2	1.94	-0.41	1.33

**Figure 4:**
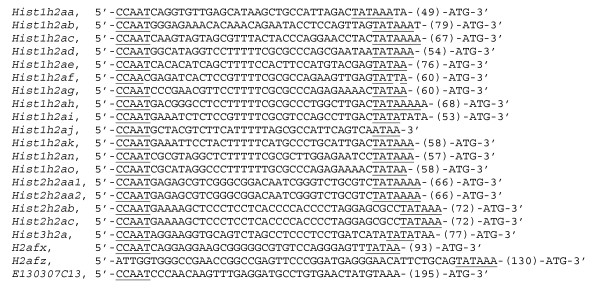
Sequences between CCAAT and TATA boxes upstream of the histone H2a and *E130307C13 *coding regions. Underlines indicate CCAAT and TATA boxes. The ATGs located at the 3'-end indicate translation start codon. Numbers in parentheses represent numbers of bases not shown. The *Hist1h2aj *is a pseudogene, lacking a start codon.

Upstream of the 5'-end of *E130307C13*, no TATA box was found. In addition, the first CCAAT box lies 230 bases upstream of the translation start codon (Fig. 4). The other replication-dependent H2a genes have the first CCAAT and TATA boxes within 100 bases upstream of the translation start codon (Fig. 4). The replication-independent genes *H2afx *and *H2afz *also have a TATA box (Fig. 4). Thus, histone H2a genes have a TATA box in the promoters, except for *E130307C13*. Interestingly, the promoter of *H2afz *lacks CCAAT box but includes TATA box, on the other hand, that of *E130307C13 *lacks TATA box but includes CCAAT box (Fig. 4).

**Figure 5:**
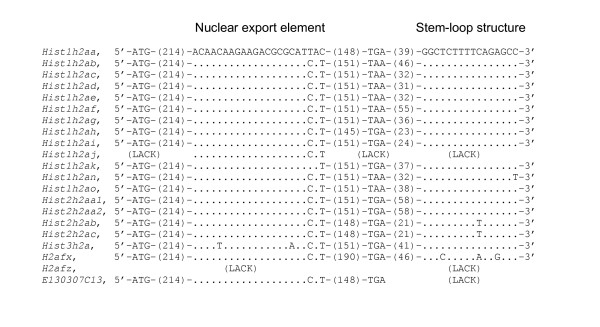
Alignment of nuclear export elements and stem-loop structures. ATG indicates translation start codon. TAA or TGA indicates translation stop codon.

In addition, the 3'-UTR of the *E130307C13 *mRNA does not include the conserved stem-loop structure (Fig. 5). But the *E130307C13 *mRNA has two poly (A) signals at the middle and near the 3'-end. It indicates that the *E130307C13 *gives rise to two differentially polyadenylated mRNA transcripts. Considering the position at the 3'-UTR, it has a possibility that the poly (A) signal near the 3'-end is functional. Except for the pseudogene *Hist1h2aj*, the replication-dependent H2a mRNAs have the conserved stem-loop structure at the 3'-UTR. *H2afx *gives rise to a cell-cycle-regulated mRNA ending in the stem-loop when it is transcribed during S-phase, and a polyadenylated mRNA that is present in G1-phase cells [2,8]. *H2afz *mRNA lacks the stem-loop structure and has poly (A) signals. *H2afz *contains four introns in the protein-coding region and needs a splicing mechanism to produce the mature mRNA. Interestingly, *E130307C13 *lacks introns. Comparing the transcript structure of *E130307C13 *with those of the other histone H2a genes suggests that *E130307C13 *has replication-independent characteristics.

## Conclusion

The bioinformatics imply that *E130307C13 *is a replication-independent H2a gene. In addition, quantitative RT-PCR analysis shows that it is replication-independent. Thus, it is *H2afj*, a novel replication-independent H2a gene in mouse.

## Methods

### Phylogenetic tree construction

Multiple alignment of 44 amino acids sequences of histone H2a related proteins from human and mouse was created using the CLUSTAL W [12] on the DNA Databank of Japan. The phylogenetic tree by the neighbor-joining method with 1000 bootstrap analyses was constructed based on the multiple alignment using MEGA version 2.1 [13].

### Cell cycle synchronization

The cell cycle of Hepa 1–6 cells was synchronized at the end of G1-phase by the addition of thymidine-hydroxyurea. The cell cycle arrest was released by washing out the thymidine-hydroxyurea, then the cells were harvested at intervals of 1 h from 0 h to 11 h.

### RNA extraction

Total RNA was extracted by using the RNeasy mini kit (Qiagen) according to the manual for the cell line. After that, each sample was treated with DNase I.

### cDNA synthesis

RNA (approximately 0.5 μg) and random hexamer primers were heated to 70°C for 10 min, followed by cooling on ice for 5 min. The cDNA was synthesized using Superscript III First Strand buffer (Invitrogen) according to the manual. The reverse transcriptase was inactivated by a 15-min incubation at 70°C.

### Quantitative PCR

The following primers were used: 5'-AACTGTAGCCCGGCCCG-3' and 5'-TTCGTCTGTTTGCGCTTT-3' (primer set 1, product size 100 bp) and 5'-CAACAAGCTGCTGGGCAAA-3' and 5'-TCGCCTTATGGTGGCTCTCC-3' (primer set 2, product size 101 bp) for transcripts of *Hist2h2aa2*; 5'-ACTCCGGAAAGGCCAAGACA-3' and 5'-GTTGTCCTAGATTTCAGGTG-3' for *H2afz*, product size 100 bp; 5'-CGTCCTGCCCAATATCCAG-3' and 5'-TCTGCACCCGTCTGTCG-3' (primer set 1, product size 90 bp) and 5'-AAGCAGGGCGGTAAGGTG-3' and 5'-TCCGCGTAGTTGCCCTTC-3' (primer set 2, product size 110 bp) for *E130307C13*; and 5'-TGTGTCCGTCGTGGATCTGA-3' and 5'-CCTGCTTCACCACCTTCTTGA-3' for *GAPDH *(glyceraldehyde-3-phosphate dehydrogenase), product size 76 bp. Quantification of GAPDH mRNA was used as a control for data normalization. PCR amplification was performed on an ABI PRISM 7700 Sequence Detection System (Applied Biosystems). The PCR conditions were an initial step of 30 s at 95°C, followed by 40 cycles of 5 s at 95°C and 30 s at 60°C. The SYBR premix Ex *Taq *(Takara) was used according to the manual. Expression was assessed by evaluating threshold cycle (C_T_) values. The relative amount of expressed RNA was calculated using Livak and Schmittgen's method [14].

## Authors' contributions

HN designed this study, carried out the molecular biological studies, and the molecular evolutionary studies. TS and YT carried out synchronization of cells and quantitative RT-PCR. YH participated in the design of this study.
